# Outcomes of elderly patients treated with chemoradiotherapy for anal squamous cell carcinoma: a multicenter study of the German cancer consortium - radiation oncology group (DKTK-ROG)

**DOI:** 10.1016/j.ctro.2026.101278

**Published:** 2026-09-03

**Authors:** D. Martin, F. Rödel, G. Kalinauskaite, I. Tinhofer, D. Zips, C. Gani, T. Schimek-Jasch, H. Schäfer, A.L. Grosu, E. Thomas, M. Krause, H. Dapper, S. Combs, C. Hoffmann, M. Stuschke, F. Walter, C. Belka, I. Kurth, W.W. Hadiwikarta, M. Baumann, C. Rödel, E. Fokas

**Affiliations:** a Department of Radiotherapy and Oncology, University Hospital, Goethe University, Frankfurt, Germany; b Frankfurt Cancer Institute (FCI), Frankfurt, Germany; c German Cancer Consortium (DKTK), Partner Site Frankfurt, and German Cancer Research Center (DKFZ), Heidelberg, Germany.; d Department of Radiation Oncology, Charité-University Hospital Berlin, Berlin, Germany.; e German Cancer Consortium (DKTK), Partner Site Berlin, and German Cancer Research Center (DKFZ), Heidelberg, Germany.; f Department of Radiation Oncology, Faculty of Medicine, University Hospital Tübingen, Eberhard Karls University Tübingen, Tübingen, Germany; g German Cancer Consortium (DKTK), Partner Site Tübingen, and German Cancer Research Center (DKFZ), Heidelberg, Germany; h Department of Radiation Oncology, Medical Center, Faculty of Medicine, University of Freiburg, Germany; i German Cancer Consortium (DKTK), Partner Site Freiburg, and German Cancer Research Center (DKFZ), Heidelberg, Germany; j OncoRay - National Center for Radiation Research in Oncology, Faculty of Medicine and University Hospital Carl Gustav Carus, TU, Dresden, Helmholtz-Zentrum Dresden – Rossendorf, Germany; k Department of Radiotherapy and Radiation Oncology, Faculty of Medicine and University Hospital Carl Gustav Carus, TU, Dresden, Germany; l Helmholtz-Zentrum Dresden - Rossendorf, Institute of Radiooncology – OncoRay, Dresden, Germany; m National Center for Tumor Diseases (NCT), Partner Site Dresden, Germany: German Cancer Research Center (DKFZ), Heidelberg, Germany; Faculty of Medicine and University Hospital Carl Gustav Carus, Technische Universität Dresden, Dresden, Germany, and; Helmholtz Association / Helmholtz-Zentrum Dresden - Rossendorf (HZDR), Dresden, Germany; n German Cancer Consortium (DKTK), Partner Site Dresden, and German Cancer Research Center (DKFZ), Heidelberg, Germany; o Department of Radiation Oncology, Technical University Munich (TUM), Munich, Germany; p German Cancer Consortium (DKTK), Partner Site Munich, and German Cancer Research Center (DKFZ), Heidelberg, Germany; q Department of Radiotherapy, University Hospital Essen, University of Duisburg-Essen, Essen, Germany; r German Cancer Consortium (DKTK), Partner Site Essen, and German Cancer Research Center (DKFZ), Heidelberg, Germany; s Department of Radiation Oncology, University Hospital LMU Medizin, Ludwig-Maximilians-Universität München, Germany; t German Cancer Research Center (DKFZ) and German Cancer Consortium (DKTK) Core Center, Heidelberg, Germany; u Department of Radiation Oncology, Cyberknife and Radiation Therapy, Faculty of Medicine and University Hospital of Cologne, University of Cologne, Cologne, Germany; Center for Integrated Oncology (CIO), University Hospital of Cologne, Faculty of Medicine and University of Cologne, Cologne, Germany.

**Keywords:** Anal cancer, Chemoradiotherapy, Elderly, Outcome, Multicenter cohort

## Abstract

**Background:**

Older patients with anal squamous cell carcinoma (ASCC) are underrepresented in prospective studies evaluating chemoradiotherapy (CRT). We evaluated patient−/tumor−/treatment characteristics and oncologic outcomes in older patients treated with CRT in a large retrospective multicenter cohort study of the DKTK-ROG.

**Materials and Methods:**

Patients with localized ASCC treated with CRT between 2004 and 2018, were retrospectively analyzed. Older patients were defined as age ≥ 70 years at CRT initiation. Freedom from recurrence (FFR) and overall survival (OS) were analyzed using Kaplan-Meier estimates, competing-risk analyses, and Cox regression models.

**Results:**

A total of 683 patients were analyzed, including 180 older patients (≥70 years, 26%). The 3-year FFR and OS rates for the entire cohort were 80.0% (95% CI, 76.9–83.3%) and 87.1% (95% CI 84.5–89.9%), respectively. Competing risk regression for locoregional failure including non complete response or distant metastases with death as competing risk revealed no significant difference between both groups (*p* = 0.5, *p* = 0.9, respectively). In contrast, older patients demonstrated inferior OS (*p* < 0.001). In multivariable analysis, advanced age remained associated with inferior OS (HR 2.51, 95% CI 1.73–3.64, *p* < 0.001), whereas no association between older age and FFR was observed (HR 1.04, 95% CI 0.69–1.58, *p* = 0.856); advanced T category and nodal involvement remained the dominant prognostic factors for worse FFR.

**Interpretation:**

Definitive CRT provides meaningful oncologic control in selected older patients with localized ASCC. Chronological age alone should not preclude curative-intent treatment. Future studies should integrate geriatric assessment to better individualize treatment strategies in older patients.

## Highlights

• Elderly (≥ 70 years) patients with anal cancer can be treated with curative intent chemoradiotherapy.
• Age does not predict recurrence after chemoradiotherapy.
• Older patients have lower survival but similar cancer control.
• Treatment decisions should not rely only on patient age.

## 1 Introduction

The incidence of anal squamous cell carcinoma (ASCC) has increased in recent years across all age groups, including elderly patients, despite it remaining a relatively rare malignancy that accounts for approximately 2–4% of gastrointestinal cancers [1], [2]. The standard of care for non-metastatic ASCC remains definitive chemoradiotherapy (CRT) [3]. As the global population continues to age, the management of ASCC in elderly patients has become increasingly relevant. Older patients frequently present with comorbidities, reduced physiologic capacities, impaired functional status, and increased vulnerability to treatment-related toxicity. In addition, elderly patients are substantially underrepresented in prospective chemoradiotherapy trials, limiting the evidence base guiding treatment decisions in this population [4]. Furthermore, available evidence regarding CRT in elderly patients with ASCC is largely derived from relatively small single-center series with heterogeneous treatment approaches and inconsistent definitions of “elderly” patients. As such, the importance of distinguishing between chronological age and frailty to enable individualized treatment strategies in older cancer patients has been recently emphasized [4]. This lack of evidence highlights the need for further research to inform treatment decisions and improve outcomes for elderly patients with ASCC. Therefore, we analyzed a large multicenter cohort from the German Cancer Consortium Radiation Oncology Group (DKTK-ROG) from 8 tertiary cancer centers to evaluate patient, tumor-, and treatment characteristics and oncologic outcomes of older patients treated with definitive CRT for localized ASCC.

## 2 Materials and methods

### 2.1 Patients and treatment

Patients were treated with 5-FU/MMC-based CRT between 2004 and 2018 and data were collected as part of a retrospective data collection which was initiated in 2018 and therefore patients that were treated after 2018 could not be included. Capecitabine-based chemotherapy was permitted according to institutional standards. Radiotherapy techniques included three-dimensional conformal radiotherapy (3D-CRT) and intensity-modulated radiotherapy (IMRT/VMAT), depending on treatment period and institutional practice, as described before [5]. Inclusion criteria were histologically proven ASCC, cT1–4 cN_any_M0, CRT with curative intent, and a minimal cumulative dose to the tumor of 40 Gy (EQD2). The dose cut-off of 40 Gy was chosen because this seems to be the minimal dose needed for curative intent treatment for smaller tumors [6]. A total of 693 patients were identified across eight DKTK-ROG tertiary cancer centers. After exclusion of patients with missing follow-up, 683 patients remained for analysis. This study was approved by the ethical committees of all DKTK-ROG partner sites (Medical Faculty of Frankfurt University, Ethics committee, Centralized protocol number 458/17). Older patients were defined as being 70 years of age or older at the initiation of CRT. The age cut-off of 70 years was selected in accordance with several previous studies in geriatric oncology and anal cancer literature [7], [8], [9].

### 2.2 Statistical analysis

Patient data were collected in the RadPlanBio infrastructure of the DKTK [10]. Survival times were calculated from start of CRT to the date of respective events or last follow-up. The patients and tumor variables evaluated included TN-subcategories, age, gender, Karnofsky Peformance Score (KPS), HIV-status, and baseline blood values including white blood cell (WBC), platelets and hemoglobin. Lack of clinical complete response (cCR) at restaging or any locoregional recurrence during follow-up after initial cCR were counted as events when assessing local failure. Time-to-event was calculated from start of radiotherapy to the date of the respective event or last follow-up visit. Cumulative incidences of local failure and distant metastasis were calculated with death from any cause as competing risk. Overall survival (OS) was calculated with death of any cause as the event. Freedom from recurrence (FFR) was calculated with the date of either local or locoregional failure, non-complete response, or distant metastases as event, whichever occurred first.

Missing covariates (< 10%) were imputed using Additive Regression, Bootstrapping and predictive mean matching using the R package ‘Hmisc’. The rates of missing covariates and distribution of missing variables that were imputed are summarized in **Supplementary Table 1–2**. Differences in survival were calculated using the log rank test or Cox regression analysis. The assumption of proportional hazard was verified by assessing the scaled Schoenfeld residuals. Follow up was calculated using the reverse Kaplan-Meier approach. Finally, overall treatment time (OTT) was defined as the interval between the first and last day of radiotherapy. OTT was compared between both groups and additionally analyzed using predefined thresholds (>42, >45, and > 49 days), reflecting commonly used definitions of prolonged treatment duration in anal cancer CRT series. Associations between OTT and oncologic outcomes were evaluated using univariable and multivariable Cox proportional hazards regression models. All statistical analysis was performed with R (Version 4) [11]. A *p*-value ≤0.05 was considered significant.

## 3 Results

### 3.1 Patient characteristics

Patient characteristics are summarized in Table 1. From 683 patients included in the analysis, 180 were 70 years or older at initiation of treatment. There were no significant differences according to age, body mass index, T category, N category, KPS or white blood cell count, platelets or hemoglobin at baseline. The proportion of HIV positive patients was significantly higher in the <70 year group (12% vs 2%, *p* < 0.001), whereas older patients showed numerically lower rates of nodal involvement. Importantly, the majority of older patients demonstrated preserved KPS, suggesting careful selection for curative-intent CRT. Older patients had a statistically significant higher prescribed dose to the primary tumor.

**Table 1:** Patient's characteristics.

		All patients *N* = 683	Non-elderly *N* = 503	Elderly *N* = 180	*P* Value
Age, years median (IQR; range)		59 (51–70; 21–95)	55 (49–62; 21–69)	76 (72–79; 70–95)	< 0.001
Sex	male (%)	237 (35)	177 (35)	60 (33)	
	female (%)	446 (65)	326 (65)	120 (67)	0.7
HIV positive		65 (10)	62 (12)	3 (2)	< 0.001
BMI kg/m^2^		24 (21.3–27)	24 (21–27)	24.8 (22–27)	0.12
KPS	90–100	572 (84)	428 (85)	144 (80)	
	70–80	93 (14)	64 (13)	29 (16)	
	50–60	18 (2)	11 (2)	7 (4)	0.16
T-Stage	T1	118 (17)	92 (18)	26(14)	
	T2	308 (45)	228 (45)	80 (45)	
	T3	182 (27)	131 (26)	51 (28)	
	T4	75 (11)	52 (10)	23 (13)	0.54
N-Stage	N0	399 (58)	285 (57)	114 (63)	
	N+	284 (42)	218 (43)	66 (37)	0.12
Pre-treatment colostomy		48 (7)	36 (7)	12 (6.6)	1
Leukocytes (/nl)		7.61 (6.07–9.25)	7.7 (6–9.25)	7.46 (6.3–9.2)	0.9
Hemoglobin (mg/dl)		13.4 (12.2–14.2)	13.4 (12.3–14.3)	13.10 (12–14.1)	0.07
Platelets (/nl)		265 (221−320)	268 (221–325)	255 (223–304)	0.2
RT dose to Primary tumor Gy median (IQR, range)		59.4 (54.8–59.4; 44.6–68.4)	57.6 (54–59.4; 44.6–66)	59.4 (55.7–59.4; 45–68.4)	0.028

Abbreviations: HIV, human immunodeficiency virus; RT, radiotherapy; BMI, body mass index

### 3.2 Overall treatment time

OTT was significantly longer in older patients (median [IQR] 45 [43–48] vs. 44 [42–46] days, *p* = 0.002). Accordingly, prolonged OTT was more frequently observed among older patients using thresholds of >42 days (76.7% vs. 63.4%, *p* = 0.001) and > 45 days (47.2% vs. 32%, *p* < 0.001), whereas the difference for OTT >49 days showed only a trend (15.6% vs. 10.7%, *p* = 0.09, **Supplementary Table 3**). In univariable Cox regression, increasing OTT was associated with inferior OS when analyzed continuously per 7-day increase (HR 1.15, 95% CI 1.01–1.31, *p* = 0.031), although this association did not remain significant after adjustment for clinicopathologic and laboratory parameters. OTT was not independently associated with FFR (**Supplementary Table 4**).

Chemotherapy dose reductions were only available in a subset of 107/683 (15.7%) patients of which 24 were ≥ 70 years old. In this subgroup there were no significant differences in dose reductions between both groups for 5-FU and MMC, respectively (< 70 years vs. ≥ 70 years; Mean +/− SD; 5-FU: 95% +/− 14% vs. 89% +/− 21%, *p* = 0.15; MMC: 98% +/− 8% vs. 93% +/− 17%, *p* = 0.22).

### 3.3 Treatment outcome

**Fig. 1:**
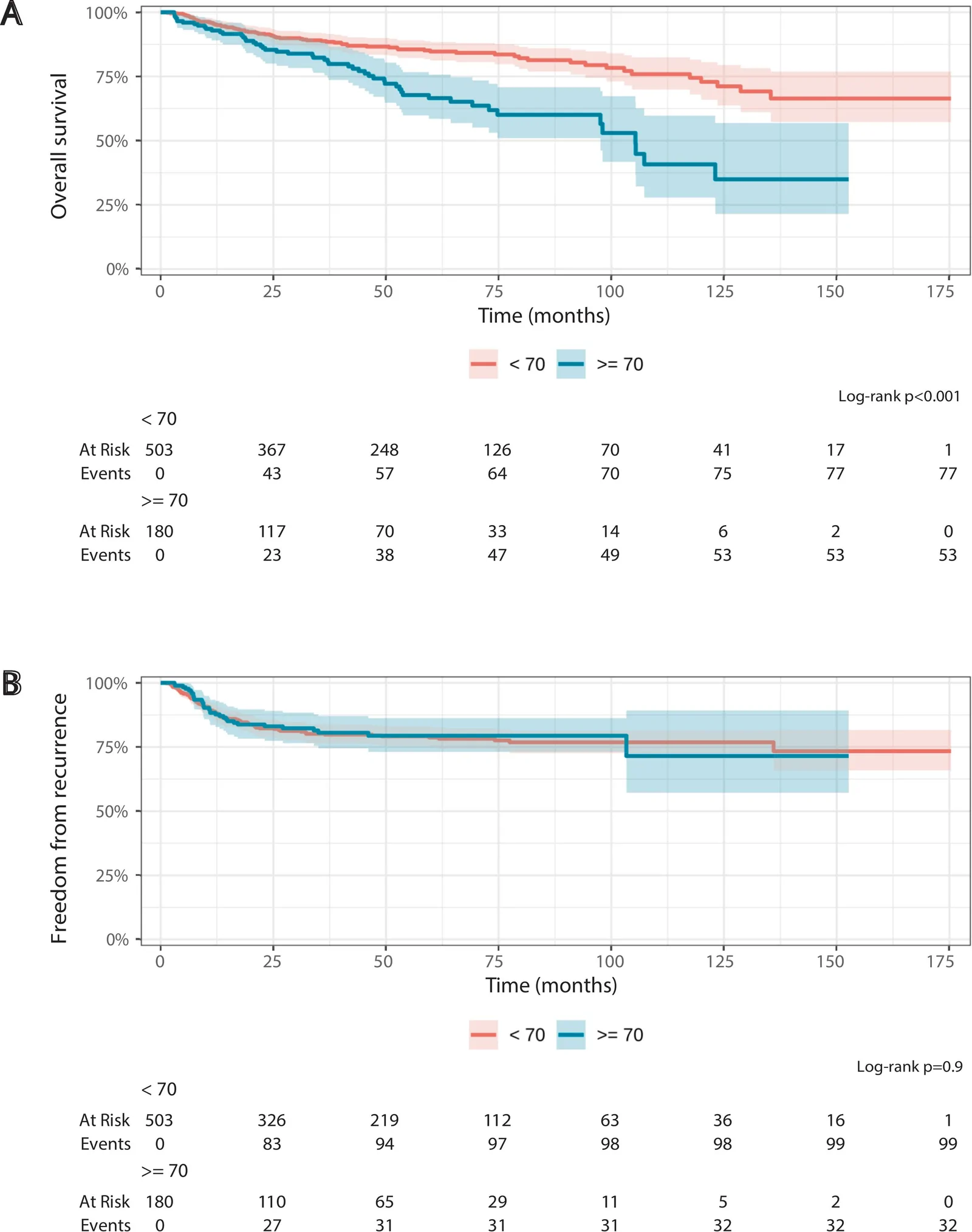
Kaplan-Meier curves demonstrating (A) overall survival (OS) and (B) freedom from recurrence (FFR) in elderly (≥70 years) versus non-elderly (<70 years) patients with localized ASCC treated with definitive chemoradiotherapy. Survival was calculated from initiation of treatment. Statistical comparisons were performed using the log-rank test.

The resulting median follow up was 56.5 months (95% CI, 51.48–60.59). The 3-year FFR and OS for all patients were 80% (95% CI, 76.9%–83.3%) and 87.1% (95% CI 84.5% - 89.9%), respectively. There were no significant differences between both groups regarding cumulative incidence of locoregional failure (*p* = 0.6), distant metastasis (*p* = 0.9), and FFR (*p* = 0.9, Fig. 1). Patients in the older group had a significantly shorter overall survival (*p* < 0.001, Fig. 1). Due to different age cut offs in the literature we also conducted the analysis using age as a continuous parameter and there was no significant association with locoregional failure (HR 0.99, 95% CI 0.98–1.01, *p* = 0.49), distant metastasis (HR 1.00, 95% CI 0.98–1.02, *p* = 0.75), FFR (HR 0.99, 95% CI 0.98–1.01,*p* = 0.4) but a significant association with OS (HR 1.04, 95% CI 1.02–1.05, *p* < 0.001).

**Fig. 2:**
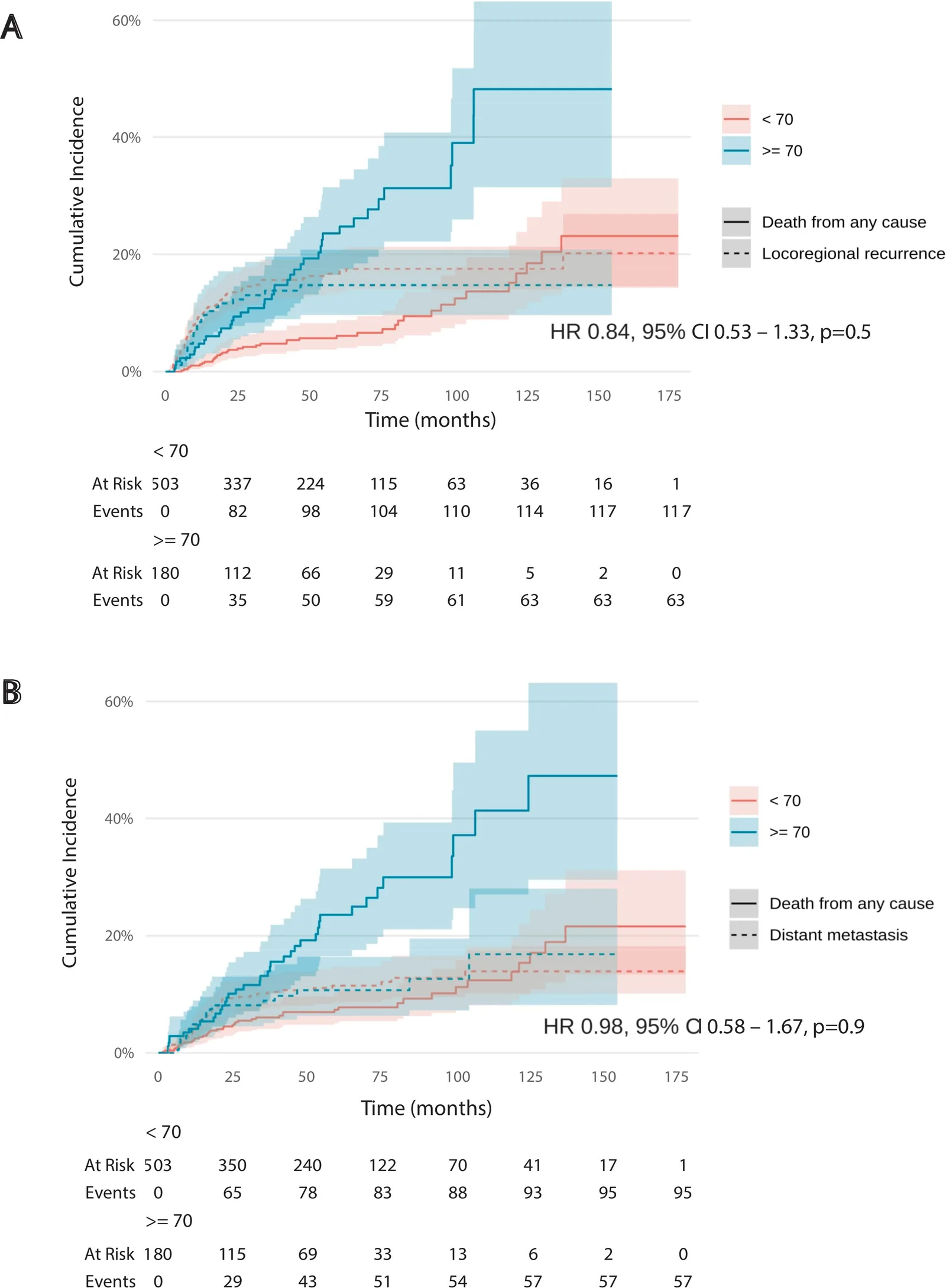
Competing risk analysis for cumulative incidence for (A) locoregional failure and (B) distant metastasis in elderly (≥70 years) and non-elderly (<70 years) patients with localized ASCC treated with definitive chemoradiotherapy. Survival was calculated from initiation of treatment. Death from any cause was treated as a competing event. Numbers at risk are displayed below each panel.

Fig. 2 shows the cumulative incidence of locoregional failure and distant metastases with death as a competing risk divided by age group. Cumulative incidence analyses demonstrated higher competing mortality in older patients, whereas there were no significant differences between incidence of locoregional failure (HR 0.84, 95% CI 0.53–1.33, *p* = 0.5) or distant metastases (HR 0.98, 95% CI, 0.58–1.67, *p* = 0.9) in competing risk regression analysis. In a sensitivity analysis, we also did a competing risk regression using age as a continuous variable and found also no significant effect on locoregional failure (HR 0.99, 95% CI 0.98–1.01, *p* = 0.4) and distant metastases (HR 1.0, 95% CI 0.99–1.02, *p* = 0.9).

In multivariable Cox regression analysis for OS, older age (≥70 years) remained independently associated with inferior overall survival (HR 2.51, 95% CI 1.73–3.64, *p* < 0.001) (Table 2). Advanced T category also significantly predicted worse OS, with cT3 and cT4 tumors demonstrating significantly increased hazards compared with cT1 tumors. In addition, higher baseline hemoglobin levels were independently associated with improved OS. Finally, in multivariable Cox regression analysis for FFR (Table 3), older age lacked prognostic significance (HR 1.14, 95% CI 0.69–1.58, *p* = 0.856). In contrast, advanced T stage remained strongly associated with worse FFR, especially for cT4 tumors (HR 8.04, 95% CI 2.92–22.09, *p* < 0.001), as it was for positive nodal status (HR 1.95, 95% CI 1.33–2.86, *p* < 0.001).

**Table 2:** Uni- and multivariable Cox regression analysis for overall survival (OS).

	Univariable Analysis		Multivariable Analysis	
	**HR (95% CI)**	**p**	**HR (95% CI)**	**p**
**Age group**				
Elderly (≥70 years) vs <70 years	2.29 (1.61–3.25)	<0.001	2.51 (1.73–3.64)	<0.001
**Sex**				
Female vs male	0.73 (0.52–1.04)	0.084	0.78 (0.52–1.17)	0.23
**HIV status**				
HIV+ vs HIV−	1.21 (0.71–2.01)	0.491	1.17 (0.62–2.22)	0.623
⁎**BMI**	1.02 (0.98–1.06)	0.362	1.02 (0.99–1.06)	0.261
**T stage**				
T1	ref		ref	
T2	1.98 (1.03–3.79)	0.04	1.78 (0.92–3.46)	0.087
T3	3.31 (1.72–6.37)	<0.001	2.56 (1.27–5.16)	0.008
T4	3.91 (1.85–8.3)	<0.001	2.52 (1.09–5.84)	0.030
**N stage**				
N+ vs N0	1.65 (1.17–2.33)	0.005	1.18 (0.81–1.73)	0.374
**KPS**				
90–100	ref		ref	
70–80	2.35 (1.55–3.57)	< 0.001	2.01 (1.30–3.12)	0.002
50–60	2.99 (1.39–6.45)	0.005	2.00 (0.85–4.71)	0.11
⁎**Platelets (/nl)**	1.00 (0.99–1.00)	0.104	0.99 (0.99–1.00)	0.886
⁎**Hemoglobin (g/dL)**	0.87 (0.79–0.94)	<0.001	0.91 (0.84–0.99)	0.045
⁎**White blood cells (/nl)**	1.06 (1.02–1.11)	0.004	1.04 (0.99–1.09)	0.122

Hazard ratios (HRs) and corresponding 95% confidence intervals (CIs) were calculated using Cox proportional hazards regression models. BMI, body mass index; HIV, human immunodeficiency virus; HR, hazard ratio; KPS, Karnofsky performance score.

⁎ Parameters were included as continuous variables.

**Table 3:** Uni- and multivariable Cox regression analysis for freedom from recurrence (FFR).

	Univariable Analysis		Multivariable Analysis	
	HR (95% CI)	p	HR (95% CI)	p
**Age group**				
Elderly (≥70 years) vs <70 years	0.96 (0.65–1.43)	0.85	1.04 (0.69–1.58)	0.856
**Sex**				
Female vs male	0.69 (0.49–0.99)	0.041	0.78 (0.53–1.16)	0.22
**HIV status**				
HIV+ vs HIV−	1.22 (0.71–2.09)	0.491	1.25 (0.67–2.29)	0.489
⁎**BMI**	1.00 (0.97–1.04)	0.827	1.01 (0.98–1.06)	0.462
**T stage**				
T1	ref		ref	
T2	4.30 (1.72–10.79)	0.002	3.56 (1.40–9.05)	0.008
T3	7.03 (2.79–17.69)	<0.001	4.94 (1.89–12.84)	0.001
T4	13.08 (5.06–33.81)	<0.001	8.04 (2.92–22.09)	<0.001
**N stage**				
N+ vs N0	2.94 (2.06–4.20)	<0.001	1.95 (1.33–2.86)	<0.001
**KPS**				
90–100	ref		ref	
70–80	1.77 (1.14–2.74)	0.011	1.36 (0.86–2.15)	0.19
50–60	3.33 (1.62–6.86)	0.001	2.16 (0.95–4.91)	0.07
⁎**Platelets (/nl)**	1.002 (1.001–1.004)	0.002	0.99 (0.99–1.00)	0.919
⁎**Hemoglobin (g/dL)**	0.89 (0.83–0.97)	0.006	0.98 (0.89–1.06)	0.589
⁎**White blood cells (/nl)**	1.075 (1.03–1.12)	< 0.001	1.02 (0.97–1.07)	0.433

Hazard ratios (HRs) and corresponding 95% confidence intervals (CIs) were calculated using Cox proportional hazards regression models. BMI, body mass index; HIV, human immunodeficiency virus; HR, hazard ratio; KPS, Karnofsky performance score.

⁎ Parameters were included as continuous variables.

## 4 Discussion

In this large multicenter DKTK-ROG analysis, carefully selected older patients that were treated at high volume cancer centers with definitive CRT for localized ASCC demonstrated inferior OS, whereas FFR remained largely comparable to those of younger patients. Notably, older age was not independently associated with impaired FFR in multivariable analysis, while advanced T stage and nodal involvement remained the dominant prognostic factors for worse FFR. These findings suggest that competing mortality rather than inferior oncologic disease control predominantly accounts for the observed survival differences in older patients and support individualized treatment strategies beyond chronological age alone. The negative prognostic impact of baseline hemoglobin has already been reported several times in ASCC and is most probably associated with underlying illnesses [12], [13], [14]. Additionally, there was a statistically significant difference for the prescribed radiotherapy dose to primary tumor but in absolute terms this difference accounts for about one fraction of radiotherapy which we do not deem to be clinically relevant, especially as these doses are all at the higher end of the spectrum for curative intent radiotherapy.

The endpoint FFR was chosen in this analysis in order to reflect as comprehensibly as possible on all kinds of treatment failure (locoregional relapse, incomplete response, distant metastasis) while excluding death from possibly non-cancer related causes. This approach was considered because in an analysis focusing on older patients competing mortality risks may substantially influence more conventional endpoints like disease free survival (DFS). However, we acknowledge that this differs from more commonly used endpoints that include death as an event and therefore direct comparisons between rates across different studies should be approached with caution.

Data about the treatment of older patients with ASCC are rare and additionally several cut-offs to define older patients have been used in the literature. Our findings are consistent with previous retrospective studies suggesting that older patients with ASCC may achieve meaningful disease control if they are fit to undergo curative intent CRT despite increased competing mortality. Additionally, we did several sensitivity analysis using age as a continuous variable in cox regression analysis and competing risk analysis which have confirmed our initial results. The currently available studies are summarized in Table 4. Recent analyses, including the Brazilian cohort reported by Camandaroba et al., similarly demonstrated comparable cancer-specific outcomes between older and younger patients despite differences in comorbidity burden and overall survival [7], [9], [15], [16], [17]. A National Cancer Database (NCDB) analysis used the age of 65 as a cut off and found that there was significantly less use of standard chemotherapy in older patients [18]. Several small retrospective series showed that CRT is feasible with patients that are older than 70 years [7], [9], [15], [17], [19], [20] although one study found that a significantly higher amount of older patients did not complete treatment, but this did not reflect in diminished oncological outcome. Dale et al. reported on a cohort of 35 patients with more than 80 years of age and showed that 19/35 patients were able to receive chemotherapy [21]. One small study showed that patients aged 65 years and older had worse outcomes but one has to keep in mind that the patients in this study were treated between 1980 and 1990 [22].

**Table 4:** Overview of the available evidence for (C)RT of anal cancer. If studies compared elderly patients with non-elderly patients only the number of elderly patients were mentioned in the table.

**Author**	**Design**	**N**	**N elderly**	**Age cut off, years**	**Treatment**	**Main survival endpoint**	**Conclusion**
**De Gara**[22]	**Retrospective, single center**	**65**	**38**	65	(C)RT	Crude disease free rate	Elderly patients with worse outcome
**Fallai [15]**	**Retrospective, single center**	**62**	**62**	70	(C)RT	Locoregional control	No significant differences
**Lestrade**[19]	**Retrospective, single center**	**76**	**76**	70	(C) RT + Brachytherapy	Local control, Distant control, OS	Feasible
**Foo**[17]	**Retrospective, single center**	**278**	**93**	70	(C)RT	OS, DFS, Local control	More treatment breaks, less chemotherapy, no differences in outcome
**Dale**[21]	**Retrospective, single center**	**213**	**35**	80	(C)RT	Local control, CSS, OS	feasible
**Raphael**[20]	**Retrospective, multicentric**	**1125**	**312**	70		OS, CSS, CFS	Less likely to complete crt
**Miller**[18]	**NCDB anaysis**	**26,796**	**9156**	65		OS	Elderly patients were less likely to receive standard of care chemoradiotherapy
**FFCD ANABASE**[16]	**Prospective, multicentric**	**1015**	**202**	75		OS, RFS, CFS	No differences
**Anczykowski**[9]	**Retrospective, single center**	**160**	**42**	70	(C)RT	OS, PFS, Locoregional control, Distant control	Less chemotherapy, more toxicity, Worse OS, PFS. Same locoregional and distant control
**Camandaroba**[7]	**Retrospective, single center**	**269**	**59**	70	(C)RT	OS, DFS	No difference in toxicity, similar outcome

Abbreviations: HIV, human immunodeficiency virus; OS, overall survival; DFS, disease free survival; PFS, progression free survival; CSS, cancer specific survival; RFS, recurrence free survival.

The biggest and most recent analysis is from the FFCD-Anabase prospective multicenter cohort and included 202 patients ≥75 years. While the use of chemotherapy and inguinal irradiation was significantly lower, there were no differences in oncological outcomes [16]. In line to our study, the proportion of HIV+ patients was significantly lower in the older cohort. Recently, preliminary results of the randomized ACT 4 trial in early stage ASCC were published, with an exploratory post-hoc analysis using an age of 70 years as a cut off showing more radiotherapy interruptions in the control arm but without any impact of the rate of clinical complete remission [6].

Importantly, chronological age alone does not adequately reflect physiologic reserve. Recent geriatric oncology literature increasingly emphasizes the importance of formal geriatric assessment tools, such as G8 screening, to guide individualized treatment decisions. The observed KPS in our older patient cohort is similar to the younger patients and suggests a selection toward fitter patients considered suitable for definitive CRT [4].

All patients included in this analysis were treated as part of the routine clinical practice at their respective institutions. Although formal geriatric assessments and comorbidity indices were not available, the interplay of age, frailty and comorbidities were likely evaluated informally by the treating clinicians, as reflected by the relatively favorable KPS observed in the cohort.

Relevant to that, older patients demonstrated modestly prolonged OTT compared with younger patients, however, this was not independently associated with worse FFR in our series. These observations suggest that limited treatment prolongation in selected older patients may not necessarily compromise oncologic disease control.

There are several limitations to the current analysis. First, the retrospective nature of the cohort could lead to selection bias. Second, there is no information regarding the comorbidities and concomitant medication of the patients available. CRT compliance represents a critical determinant of outcome in ASCC, whereas older patients are particularly vulnerable to treatment interruptions, chemotherapy reductions, and prolonged overall treatment time due to toxicity and comorbidity that adversely impact outcome [23]. Also, information on CRT tolerability and compliance was only available in a small minority of the DKTK ROG cohort, precluding robust assessment of its prognostic impact in older patients, which is another limitation of our study. Fourth, only patients that have received chemotherapy were included in the initial cohort, leaving out all patients that were deemed unfit for standard treatment according to local practice. Fifth, only patients who received at least an EQ.D2 of 40 Gy were included in this analysis, precluding patients who stopped treatment before reaching this threshold. In fact this threshold led to the exclusion of 3 patients, of whom 2 were non-elderly. Sixth, information on frailty was unavailable in our DKTK ROG cohort as geriatric assessment was not routinely performed outside clinical trials and information on HPV status was not available for the vast majority of patients. Another limitation is that cause-specific mortality data is not available for this cohort and that patients that were treated with radiotherapy only were not included due to the inclusion criteria of the initial data collection.

In conclusion, definitive CRT is feasible in carefully selected older patients with ASCC that are treated at high volume tertiary cancer centers and achieves meaningful oncologic outcomes despite increased competing mortality. Chronological age alone should not preclude curative-intent treatment. Future prospective studies integrating geriatric assessment as part of the screening and stratification process are warranted to optimize individualized treatment strategies in older patients with ASCC. Additionally, repeated assessment at predefined timepoints during and after treatments will be useful.

## Declaration of competing interest

The authors state that there are no potential conflicts of interest.
