# Supplementary material for: Outcomes of elderly patients treated with chemoradiotherapy for anal squamous cell carcinoma: a multicenter study of the German cancer consortium - radiation oncology group (DKTK-ROG)
Source: Clin Transl Radiat Oncol. 2026 Sep 3;61:101278. doi: 10.1016/j.ctro.2026.101278 (PMC13571947; doi:10.1016/j.ctro.2026.101278)
Supplement: Supplementary file 1 [file mmc1.docx]

**Supplementary Table 1.** Percentage of missing values of imputed variables

| **Variable** | **Missing cases** | **Missing percentage** |
| --- | --- | --- |
| Baseline Hemoglobin levels | 36 | 5.3% |
| Baseline Platelet levels | 39 | 5.7% |
| Baseline white blood cell count | 39 | 5.7% |
| Body Mass Index (BMI) | 68 | 9.9% |
| Karnofsky performance Score (KPS) | 63 | 9.2% |

**Supplementary Table 2.** Missingness pattern of imputed variables

| **N patients** | **Hb** | **Platelets** | **WBC** | **KPS** | **BMI** | **N missing** |
| --- | --- | --- | --- | --- | --- | --- |
| 542 | Present | Present | Present | Present | Present | 0 |
| 47 | Present | Present | Present | Present | Missing | 1 |
| 43 | Present | Present | Present | Missing | Present | 1 |
| 12 | Present | Present | Present | Missing | Missing | 2 |
| 2 | Present | Missing | Missing | Present | Present | 2 |
| 1 | Present | Missing | Missing | Missing | Missing | 4 |
| 22 | Missing | Missing | Missing | Present | Present | 3 |
| 7 | Missing | Missing | Missing | Present | Missing | 4 |
| 6 | Missing | Missing | Missing | Missing | Present | 4 |
| 1 | Missing | Missing | Missing | Missing | Missing | 5 |

WBC, white blood cell count; KPS, karnofsky performance score; BMI, body mass index; Hb, hemoglobin;

**Supplementary Table 3.** Overall treatment time (OTT) according to age group

|  | **<70 years** | **≥70 years** | **p-value** |
| --- | --- | --- | --- |
| **Overall treatment time (days)** |  |  |  |
| Median (IQR) | 44 (42–46) | 45 (43–48) | 0.002 |
| Mean ± SD | 44.2 ± 7.27 | 45.7 ± 7.75 |  |
| **Prolonged OTT** |  |  |  |
| >42 days | 319/503 (63.4%) | 138/180 (76.7%) | 0.001 |
| >45 days | 161/503 (32%) | 85/180 (47.2%) | <0.001 |
| >49 days | 54/503 (10.7%) | 28/180 (15.6%) | 0.11 |

**Supplementary Table 4.** Overall treatment time (OTT) association with clinical outcomes

| **Variable*** | **Univariable HR (95% CI)** | **p** | **Multivariable HR (95% CI)** | **p** |
| --- | --- | --- | --- | --- |
| **Overall survival (OS)** |  |  |  |  |
| OTT per 7-day increase | 1.15 (1.01–1.31) | 0.031 | 1.04 (0.92–1.18) | 0.549 |
| OTT >42 days | 1.19 (0.81–1.73) | 0.37 | 1.08 (0.73–1.59) | 0.715 |
| OTT >45 days | 1.32 (0.93–1.87) | 0.123 | 1.18 (0.82–1.69) | 0.374 |
| OTT >49 days | 1.17 (0.73–1.77) | 0.51 | 1.05 (0.65–1.68) | 0.848 |
| **Freedom from recurrence (FFR)** |  |  |  |  |
| OTT per 7-day increase | 1.1 (0.96–1.27) | 0.179 | 1.03 (0.90–1.18) | 0.655 |
| OTT >42 days | 1.6209 (0.75–1.57) | 0.66 | 0.99 (0.68–1.45) | 0.956 |
| OTT >45 days | 1.14 (0.80–1.59) | 0.47 | 1.02 (0.71–1.46) | 0.92 |
| OTT >49 days | 1.23 (0.76–2.01) | 0.407 | 1.12 (0.68–1.84) | 0.648 |

***** Comparison of OTT between elderly (≥70 years) and non-elderly (<70 years) patients and Cox regression analyses evaluating the association between OTT and oncologic outcomes. Hazard ratios (HRs) and corresponding 95% confidence intervals (CIs) were calculated using Cox proportional hazards regression models adjusted for age group, gender, HIV status, BMI, T stage, nodal status, Karnofsky performance score, baseline white blood cell count, platelet count, and hemoglobin levels. OTT, overall treatment time; OS, overall survival; FFR, freedom from recurrence; IQR, interquartile range; SD, standard deviation.
